# From ‘Memory Assessment Please’ to Masterpieces: Refining the Referral Process

**DOI:** 10.1192/bjo.2025.10410

**Published:** 2025-06-20

**Authors:** Donncha Mullin, Amy Lindsay, Helen Smith, Lorraine Mitchell, Imogen Smith

**Affiliations:** 1 NHS Lothian, Livingston, United Kingdom; 2 University of Edinburgh, Edinburgh, United Kingdom; 3 Alzheimer Scotland Dementia Research Centre, Edinburgh, United Kingdom

## Abstract

**Aims:** Inappropriate or incomplete referrals for memory assessment were a recurrent issue within our Old Age Psychiatry community multidisciplinary team (MDT) triage meetings. These referrals often lacked essential information, such as duration of cognitive concerns, patient consent, results of confusion screen blood tests, clarity on the presence of delirium versus long-standing cognitive concerns, ECG and imaging findings, or results of a cognitive screen. Some referrals were minimal, providing only the phrase “memory assessment please”, leading to inefficient use of resources, delays in assessment, and unnecessary correspondence with referring teams. Since memory assessments involve detailed 60-minute home visits by experienced nurses, followed by consultant evaluation, optimizing referral quality was imperative to reduce inappropriate referrals and improve service efficiency.

**Methods:** A retrospective analysis of referrals from wards and clinics to our weekly MDT triage meeting was conducted over a two-month period (July 1–August 31, 2024). Referrals were assessed for the presence of key information required for triage, including patient consent, confusion screen blood results, cognitive screen findings, imaging results, and clarity on the nature of the cognitive concern. Following this analysis, we collaborated with clinicians from Old Age Psychiatry, Liaison Psychiatry, Geriatrics, and administrative staff to design a standardized one-page referral form. The form, adapted from an existing referral template in use elsewhere in Scotland, was tailored to include all critical elements necessary for triage decisions.

**Results:** Implementing the new referral form led to a marked improvement in the completeness and quality of referral information. Key details, such as cognitive screen results and delirium assessments, were routinely included. This reduced the need to search clinical notes during MDT meetings, saving significant time for the 30–40 mental health professionals present. Additionally, there was a substantial reduction in follow-up emails and phone calls to clarify referrals. The streamlined process improved triage efficiency, decreased inappropriate referrals, and shortened waiting times for patients requiring assessment.

**Conclusion:** Introducing a standardized referral form significantly improved the quality and efficiency of referrals for memory assessments. By ensuring all essential information was provided upfront, we optimized resource use, minimized delays, and enhanced communication between teams. The referral form remains a living document, with ongoing review to ensure its continued relevance and effectiveness.

